# Clustering subtypes of breast cancer by combining immunohistochemistry profiles and metabolism characteristics measured using FDG PET/CT

**DOI:** 10.1186/s40644-021-00424-4

**Published:** 2021-09-27

**Authors:** Hyun Woo Kwon, Jeong Hyeon Lee, Kisoo Pahk, Kyong Hwa Park, Sungeun Kim

**Affiliations:** 1 Department of Nuclear Medicine, Korea University College of Medicine, Seoul, Korea; 2 Department of Pathology, Korea University College of Medicine, Seoul, Korea; 3 Department of Internal Medicine, Korea University College of Medicine, Seoul, Korea; 4 Department of Nuclear Medicine, Korea University Anam Hospital, Korea University College of Medicine, 73 Goryeodae-ro, Seongbuk-gu, 02841 Seoul, Korea

**Keywords:** Breast cancer, FDG PET/CT, Molecular subtypes, Clustering

## Abstract

**Background:**

The aim of this study was to investigate the effect of combining immunohistochemical profiles and metabolic information to characterize breast cancer subtypes.

**Methods:**

This retrospective study included 289 breast tumors from 284 patients who underwent preoperative ^18^ F-fluorodeoxyglucose (FDG) positron emission tomography/ computed tomography (PET/CT). Molecular subtypes of breast cancer were classified as Hormonal, HER2, Dual (a combination of both Hormonal and HER2 features), and triple-negative (TN). Histopathologic findings and immunohistochemical results for Ki-67, EGFR, CK 5/6, and p53 were also analyzed. The maximum standardized uptake value (SUV) measured from FDG PET/CT was used to evaluate tumoral glucose metabolism.

**Results:**

Overall, 182, 24, 47, and 36 tumors were classified as Hormonal, HER2, Dual, and TN subtypes, respectively. Molecular profiles of tumor aggressiveness and the tumor SUV revealed a gradual increase from the Hormonal to the TN type. The tumor SUV was significantly correlated with tumor size, expression levels of p53, Ki-67, and EGFR, and nuclear grade (all *p* < 0.001). In contrast, the tumor SUV was negatively correlated with the expression of estrogen receptors (*r* = − 0.234, *p* < 0.001) and progesterone receptors (*r* = − 0.220, *p* < 0.001). Multiple linear regression analysis revealed that histopathologic markers explained tumor glucose metabolism (adjusted R-squared value 0.238, *p* < 0.001). Tumor metabolism can thus help define breast cancer subtypes with aggressive/adverse prognostic features.

**Conclusions:**

Metabolic activity measured using FDG PET/CT was significantly correlated with the molecular alteration profiles of breast cancer assessed using immunohistochemical analysis. Combining molecular markers and metabolic information may aid in the recognition and understanding of tumor aggressiveness in breast cancer and be helpful as a prognostic marker.

**Supplementary Information:**

The online version contains supplementary material available at 10.1186/s40644-021-00424-4.

## Introduction

Breast cancer is the most common type of cancer among women, accounting for about 30 % of newly detected cancer cases in the United States, and is the second leading cause of death from cancer among women [1]. In recent decades, researchers have attempted to characterize the biology of breast cancer and standardize treatment to improve its prognosis [2–4]. Molecular markers are key to the classification of breast cancer subtypes, the selection of treatment modalities, and prognostic prediction. The expression of estrogen receptors (ER) and/or progesterone receptors (PR) determine the luminal type of breast cancer and the target for anti-hormonal therapy. Human epidermal growth factor receptor 2 (HER2) expression is a marker for HER2-enriched breast cancer and the target for anti-HER2 therapy. The absence of a molecular target (i.e., ER-negative, PR-negative, and HER2-negative) is regarded as a triple-negative (TN) breast cancer subtype that indicates a worse prognosis for the patient [5].

Intrinsic subtypes of breast cancer (luminal A, luminal B, HER2-enriched, and basal-like) are routinely used as a classification system. These immunohistochemistry (IHC) marker-based subtypes exhibit different gene expression profiles and molecular characteristics. The luminal A subtype has a low mutation rate overall, with significantly mutated genes that include PIK3CA, MAP3K1, GATA3, and MAP2K4. The luminal B subtype exhibits more frequent mutations of *TP53* and hypermethylation than the luminal A subtype. The HER2-enriched subtype is characterized by the high expression of HER2-related genes and a high frequency of APOBEC3B-associated mutations. The basal-like subtype is the most distinct of the breast cancer subtypes. It has a high mutation rate for TP53 and ATM and the loss of BRCA1 [2, 6–8].

In addition to the IHC-based system, gene-expression-based assays such as PAM50 are used to classify cancer subtypes. Studies have revealed a noticeable discordance between these two classification systems. Prat et al. [9] reported that over 35 % of luminal B tumors classified using PAM50 are identified as luminal A by IHC, while 28.2 and 30.4 % of clinically HER2-positive and negative tumors, respectively, are luminal B tumors. Around 10 % of basal-like tumors exhibit the overexpression of HER2 [2, 10, 11]. Therefore, a precise and easily accessible classification system is necessary to improve the management of breast cancer.

^18^ F-fluorodeoxyglucose (FDG) positron emission tomography (PET)/computed tomography (CT) can be used to visualize tumor biology based on glucose metabolism. Tumoral FDG uptake is significantly associated with prognostic factors for breast cancer, although the independent prognostic role of FDG uptake remains unclear [12]. Previous studies have reported a relationship between tumor FDG uptake and both clinicopathologic characteristics [13–15] and the molecular subtypes of breast cancer [16–19]. However, the role of glucose metabolism in classifying breast cancer is not yet fully understood.

The main purpose of this study was to analyze the correlation between glucose metabolism and immunohistochemical results for breast cancer and to investigate the combination of IHC profiles and metabolic information to characterize breast cancer subtypes.

## Materials and methods

### Patients

Consecutive patients with primary breast cancer who underwent a curative resection from March 2013 to December 2015 were included in this retrospective study. Inclusion criteria for patient selection were (1) those who were initially diagnosed with primary breast carcinoma, (2) those who underwent preoperative FDG PET/CT at our hospital, and (3) those who underwent curative surgery, including breast-conserving surgery and mastectomy, using a patient-optimized technique at our hospital. Exclusion criteria were (1) those who underwent neoadjuvant chemotherapy, (2) patients who had more than one primary malignancy, (3) those who had only ductal carcinoma in situ (DCIS) or papilloma, and (4) those whose immunohistochemistry profile from the histopathologic report was unavailable (Supplementary Fig. 1). Clinical characteristics and histopathologic results for the patients were obtained from their medical records. The study protocol was approved by the Institutional Review Board of our hospital.

### FDG PET/CT

FDG PET/CT was performed using a dedicated scanner (Gemini TF 16, Philips Medical Systems). Patients fasted for 6 h. At the time of FDG injection, their blood glucose levels were confirmed to be less than 200 mg/dL. PET/CT images were acquired 54.2 ± 12.5 min after FDG injection (approximately 5.18 MBq/kg) from the skull vertex to the upper thigh of the patients. A low-dose CT scan was acquired for attenuation correction (50 mA, 120 kVp, slice thickness of 4 mm, matrix size of 512 × 512). Emission PET images were acquired for 1 min for each bed position. Transaxial PET images were reconstructed using a 3D iterative algorithm (row action maximum likelihood algorithm) with a TOF function (3 iterations, 33 subsets, matrix size 144 × 144). All FDG PET/CT images were analyzed by two experienced nuclear medicine physicians (H.W.K and S.K) in consensus. A region of interest (ROI) with a maximal diameter of 3 cm was positioned at the point of highest intensity in the tumor. After this, the size of the ROI was modified to exclude surrounding false-positive normal tissue activity. The maximum standardized uptake value (SUV) for the tumor was calculated from the ROI in transaxial PET images using the following formula: SUV = maximum activity within the tumor (MBq/ml)/maximum injected FDG dose (MBq/kg body weight).

### Histopathological analysis

Diagnosis and comprehensive histopathological analysis of the primary breast carcinoma were conducted using surgical specimens. Histological staging was based on the Scarff Bloom Richardson classification system. The histological subtype, tumor size, and nuclear grade were determined using formalin-fixed paraffin-embedded tissue sections after staining with hematoxylin and eosin. Immunohistochemical analysis was conducted using paraffin-embedded slides with primary antibodies. The proliferation index was evaluated using Ki-67 staining. Expression levels of ER (Dako, Glostrup, Denmark), PR (Dako, Glostrup, Denmark), HER2 (Thermo Scientific, IL, USA), Ki-67 (Dako, Glostrup, Denmark), and p53 (Novocastra, UK) were determined based on the percentage of positive cancer cells, cytokeratin (CK) 5/6 (Dako, Glostrup, Denmark), and epidermal growth factor receptor (EGFR; Dako, Glostrup, Denmark).

The expression of ER and PR was considered positive if the Allred scores were equal to or higher than 3 (≥ 3) [16]. Allred scores were also used to analyze ER expression levels as a continuous variable. HER2 expression was determined using positive membrane staining of the tumor cells (+ 1: 10 %, + 2: ≤30 %, + 3: ≥30 %). Fluorescence or silver-enhanced in situ hybridization analysis was conducted to confirm HER2 positivity. For Ki-67 and p53, the upper bound value of the positive cell population was used for the analysis of continuous variables. Based on the histopathologic results, the patients were classified into four subgroups: (1) Hormonal type: ER and/or PR-positive; (2) HER2 type: HER2-positive; (3) dual positive (Dual) type: features of both Hormonal and HER2 types; and (4) TN type: neither Hormonal nor HER2 type.

### Statistical analysis

Student t-tests and one-way ANOVA were performed to compare group differences for the continuous variables. Tukey’s post-hoc analysis was employed for between-group comparisons. Chi-squared tests were used to evaluate the frequency of the variables between groups. Relationships between variables were analyzed using Pearson’s correlation coefficients. Logistic regression with stepwise selection (likelihood ratio method; entry threshold, *p* < 0.05; removal threshold, *p* > 0.1) was used to determine the effect of the molecular profile on metabolic activity. Supervised clustering of the breast cancer subtypes was analyzed using the Uniform Manifold Approximation and Projection (UMAP) package implemented in R [17], in which binary variables were transformed into 0 and 1 while continuous variables were normalized using the mean and standard deviation. Receiver operating characteristic (ROC) curves were used to evaluate the optimal cutoff values for the continuous variables. All data were analyzed using MedCalc Software v19.4.1 (MedCalc Software Ltd, Ostend, Belgium) and R v3.5.1 (https://cran.r-project.org). *P* values of less than 0.05 were considered statistically significant.

## Results

### Characteristics of the patients and tumors

A total of 289 tumors from 284 patients were included in this study, including Hormonal (*n* = 182), HER2 (*n* = 24), Dual (*n* = 47), and TN (*n* = 36) tumor subtypes (Table 1). A total of 185 specimens were acquired from breast-conserving surgery, while 88 and 16 specimens were obtained from modified radical mastectomy and skin-sparing mastectomy, respectively. The mean ± standard deviation of the patients’ age was 54.7 ± 10.8 years (range, 30–87 years). The mean difference in the patients’ age was not significant between the cancer types.

**Table 1:** Characteristics of the patients and tumors

Characteristics	Hormonal (*n* = 182)	HER2 (*n* = 24)	Dual Positive (*n* = 47)	Triple Negative (*n* = 36)	*P*
Age (y)	54.8 ± 11.0	55.6 ± 10.2	53.5 ± 9.4	55.5 ± 12.1	0.813
Histology					
IDC	150	20	44	28	0.067
ILC	14	1	1	0	
Others	8	3	2	8	
T stage					
T1	121	17	24	26	0.572
T2	54	7	21	10	
T3	6	0	2	0	
T4	1	0	0	0	
N stage					0.165
N0	109	18	31	31	
N1	56	6	11	5	
N2	13	0	4	0	
N3	4	0	1	0	
Nuclear grade					< 0.001^*^
1	77	0	5	2	
2	85	7	19	11	
3	20	17	23	23	
Stage					0.451
IA	84	15	17	23	
IB	8	0	1	1	
IIA	51	5	20	10	
IIB	20	4	4	2	
IIIA	14	0	4	0	
IIIB	1	0	0	0	
IIIC	4	0	1	0	
Operation					0.256
BCS	119	13	25	28	
MRM	52	9	19	8	
SSM	11	2	3	0	

*BCS* breast conserving surgery, *IDC* invasive ductal carcinoma, *ILC* invasive lobular carcinoma, *MRM* modified radical mastectomy, *SSM* skin-sparing mastectomy; ^*^P < 0.05

Histopathologic classification identified invasive ductal carcinoma (*n* = 242), invasive lobular carcinoma (*n* = 16), and others (carcinoma with medullary features, *n* = 4; carcinoma with neuroendocrine differentiation, *n* = 1; microinvasive carcinoma, *n* = 2; papillary carcinoma, *n* = 8; cribriform carcinoma, *n* = 1; metaplastic carcinoma, *n* = 3; mucinous carcinoma, *n* = 8; and tubular carcinoma, *n* = 4). Histopathologic classification and nuclear grade differed significantly between the cancer types (*p* = 0.035 and *p* < 0.001, respectively), while the histopathologic T and N categories and stage group were not significantly different according to the cancer type. Histopathologic results for the excluded patients are described in Supplementary Table 1.

The mean ± standard deviation of the tumor size was 1.94 ± 1.56 cm (Table 2). Forty-six (15.9 %) tumors exhibited lymphatic invasion (LI), while the number of positive ER and PR tumors was 227 (78.5 %) and 212 (78.5 %), respectively. The number of positive HER2 tumors was 71 (24.6 %, Table 2).

**Table 2:** Histopathologic and molecular characteristics of tumors in this study

Characteristics	Positive case (%) or mean ± SD
Size (cm)	1.94 ± 1.56
Lymphatic invasion	46 (15.9 %)
Venous invasion	2 (0.7 %)
Perineural invasion	10 (3.5 %)
ER	227 (78.5 %)
PR	212 (73.4 %)
HER2	71 (24.6 %)
CK5/6	127 (43.9 %)
p53^*^	27.2 ± 34.8
Ki-67^*^	19.4 ± 21.8
EGFR	44 (15.2 %)

CK5/6, cytokeratin 5/6; EGFR, epidermal growth factor receptor; ER, estrogen receptor; PR, progesterone receptor; *upper bound value of positive cell population (%)

### Correlation between tumor characteristics and glucose metabolism

**Fig. 1:**
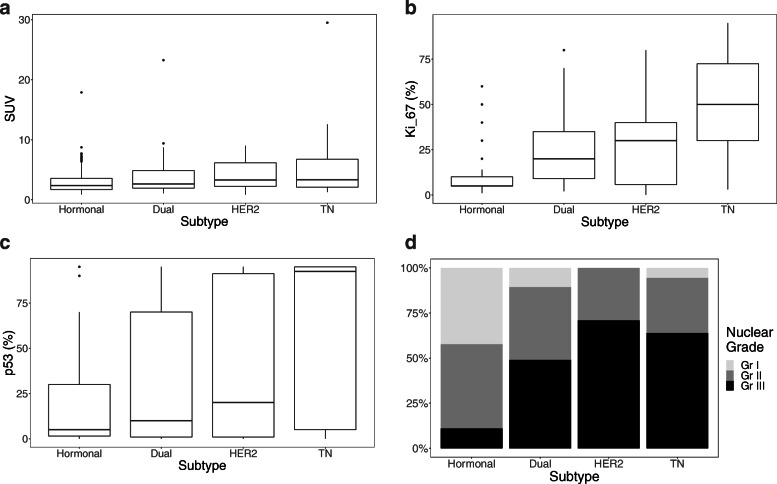
Molecular and metabolic characteristics of the breast cancer subtypes. **A** SUV, **B** Ki-67, and **C** p53 expression gradually increased from the Hormonal subtype to the TN subtype. Similarly, **D** nuclear grade III was higher for the HER2 and TN subtypes than for the other subtypes

**Fig. 2:**
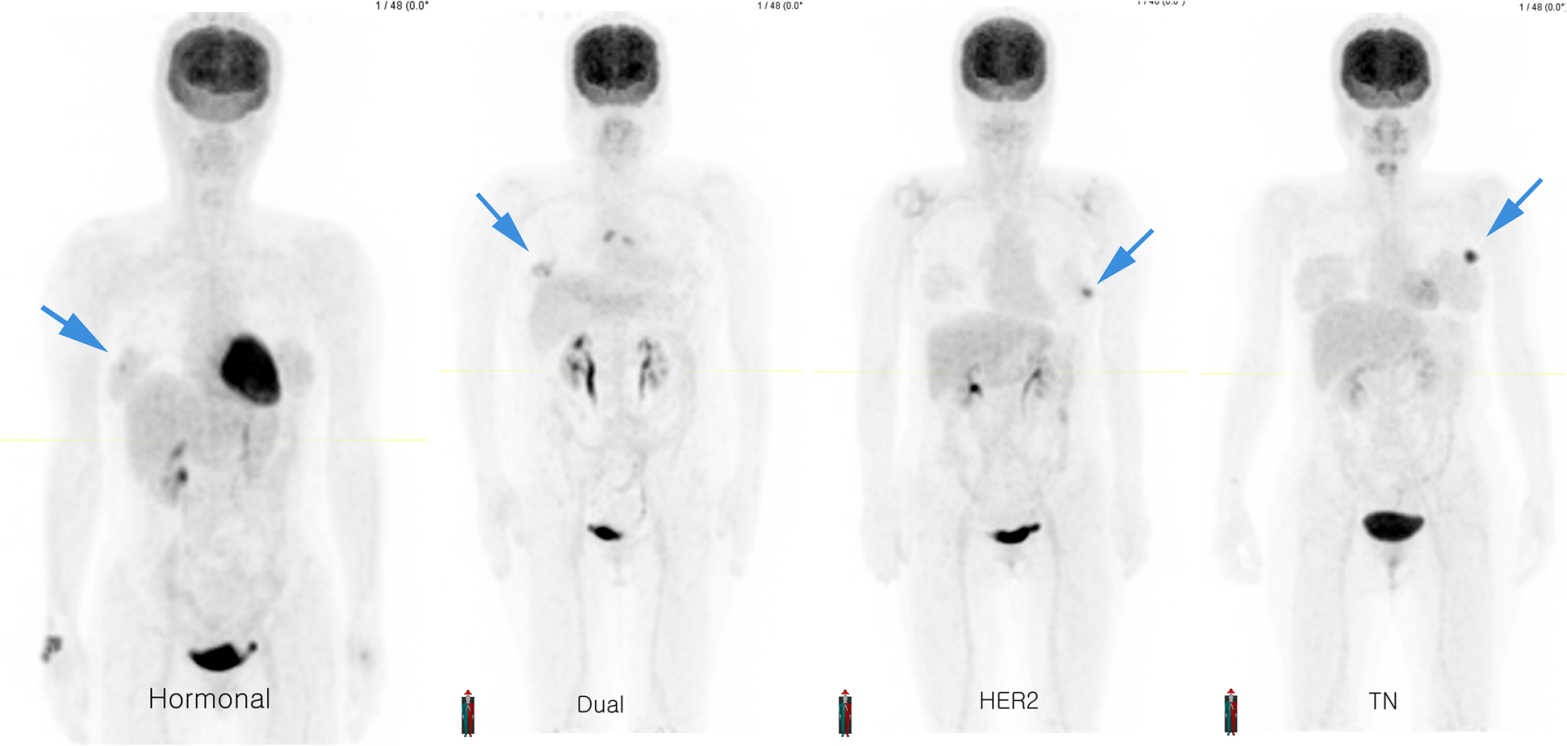
Representative FDG PET images for the breast cancer subtypes (all patients pT stage 2). In this image, the tumor SUV for the Hormonal, Dual, HER2, and TN tumor types was 2.51, 3.34, 4.88, and 8.04, respectively (blue arrow)

The mean ± standard deviation of the tumor SUV was 2.97 ± 1.99 for Hormonal, 4.18 ± 2.53 for HER2, 3.95 ± 3.61 for Dual, and 5.18 ± 5.29 for TN cancer types. Post-hoc analysis revealed that TN-type tumors showed a higher SUV than Hormonal-type tumors (*p* < 0.001). The mean SUV gradually increased from the Hormonal to the TN subtype (Figs. 1A and 2). The tumor SUV was positively correlated with the tumor size (*r* = 0.288, *p* < 0.001) and nuclear grade (r = 0.416, *p* < 0.001). Histopathologic T1 tumors (2.61 ± 1.70) exhibited a lower SUV than both T2 tumors (5.18 ± 4.20) and T3-4 tumors (5.14 ± 1.85) (both *p* < 0.001). In contrast, the tumor SUV did not differ for the N category (*p* = 0.382). Tumors with LI tended to show a higher SUV (4.25 ± 2.79) than tumors without LI (3.36 ± 3.04) (*p* = 0.068).

The tumor SUV was compared with the molecular markers monitored in this study. Tumors expressing EGFR exhibited a higher SUV (*p* < 0.001). However, tumors with HER2 tended to show a higher SUV than tumors without HER2 (*p* = 0.093). The SUV for tumors with CK 5/6 was not significantly different from that for tumors without CK 5/6 (*p* = 0.823). The SUV was significantly correlated with Ki-67 (*r* = 0.332, *p* < 0.001) and p53 (*r* = 0.247, *p* < 0.001) and negatively correlated with the expression of ER (*r* = − 0.234, *p* < 0.001) and PR (*r* = − 0.220, *p* < 0.001).

### Histopathologic and molecular profiles according to tumor subtype

The mean ± standard deviation for Ki-67 expression was 11.0 ± 12.7 for Hormonal, 26.8 ± 22.9 for HER2, 24.3 ± 19.8 for Dual, and 50.4 ± 28.4 for TN tumors (Fig. 1B, *p* < 0.001), while that for p53 expression was 17.7 ± 25.3 for Hormonal, 41.9 ± 42.6 for HER2, 230.8 ± 37.1 for Dual, and 60.8 ± 42.9 for TN tumors (Fig. 1C, *p* < 0.001). ANOVA analysis revealed that the Ki-67 expression levels in TN-type tumors were significantly higher than those in other subtypes. Ki-67 expression in HER2- and Dual-type tumors was higher than that in Hormonal-type tumors (both *p* < 0.001). Expression levels of p53 in TN tumors were also significantly higher than those in Hormonal and Dual tumors. The expression of p53 in HER2-type tumors was significantly higher than that in Hormonal-type tumors (*p* = 0.003). The proportion of nuclear grade III tumors was 11.0 % for Hormonal, 70.8 % for HER2, 248.9 % for Dual, and 63.9 % for TN tumors (*p* < 0.001, Fig. 1D). The proportion of EGFR-positive tumors was 2.2 %, 50.0 %, 10.6 %, and 63.9 % for Hormonal, HER2, Dual, and TN tumors, respectively (*p* < 0.001), while that of CK5/6-positive tumors was 43.4 %, 62.5 %, 27.7 %, and 55.6 %, respectively (*p* = 0.011). Correlations between the SUV and molecular markers were analyzed for the Hormonal subgroup. Expression levels of Ki-67 (*r* = 0.250, *p* < 0.001), expression levels of p53 (*r* = 0.308, *p* < 0.001), and nuclear grade (*r* = 0.433, *p* < 0.001) were significantly correlated with the tumor SUV.

### Analysis of molecular profiles to determine tumor metabolic activity

Univariate linear regression analysis was conducted to evaluate the significance of tumor histopathologic profiles with regards to the glucose metabolic phenotype (Table 3). Tumor size, nuclear grade, and the expression levels of Ki-67, p53, and EGFR were significant determinants of glucose metabolism. A multivariate regression model (model 1, Table 4) that included both histopathologic and immunohistochemical profiles showed that tumor size, nuclear grade, and Ki-67 were independent factors for the determination of the tumor SUV, although their explanation power was low (adjusted R^2^ of 0.238). Model 2, which was based on the immunohistochemical profiles, showed that Ki-67, p53, and EGFR were independent factors for the SUV. Interestingly, there was a significant interaction between p53 and EGFR (*p* = 0.028).

**Table 3:** Univariate linear regression analysis for determining metabolic activity

Variable	Coefficient	95 % CI	*P*
Histopathologic			
Size	0.556	0.341–0.770	< 0.001
Nuclear Grade	1.647	1.229–2.064	< 0.001
Lymphatic invasion	0.885	-0.064-1.834	0.068
Immunohistochemical			
ER	-0.216	− (0.321 − 0.112)	< 0.001
HER2	0.692	-0.115-1.500	0.093
Ki-67	0.046	0.031–0.061	< 0.001
p53	0.021	0.012–0.031	< 0.001
EGFR	1.704	0.753–2.656	< 0.001
CK 5/6	0.080	-0.623-0.784	0.823

**Table 4:** Multivariate linear regression analysis for determining metabolic activity

Model	Variable	Coefficient	95 % CI	*P*	Adjusted R^2^
1	Nuclear Grade	1.119	0.632–1.606	< 0.001	0.238
	Ki-67	0.024	0.007–0.041	0.005	
	Size	0.469	0.270–0.668	< 0.001	
2	Ki-67	0.034	0.016–0.052	< 0.001	0.126
	p53	0.015	0.003–0.028	0.018	
	EGFR	1.823	0.328–3.319	0.017	
	p53*EGFR	-0.026	− (0.049 − 0.003)	0.028	

### Clustering of breast cancer subtypes using molecular profiles and tumor metabolism

**Fig. 3:**
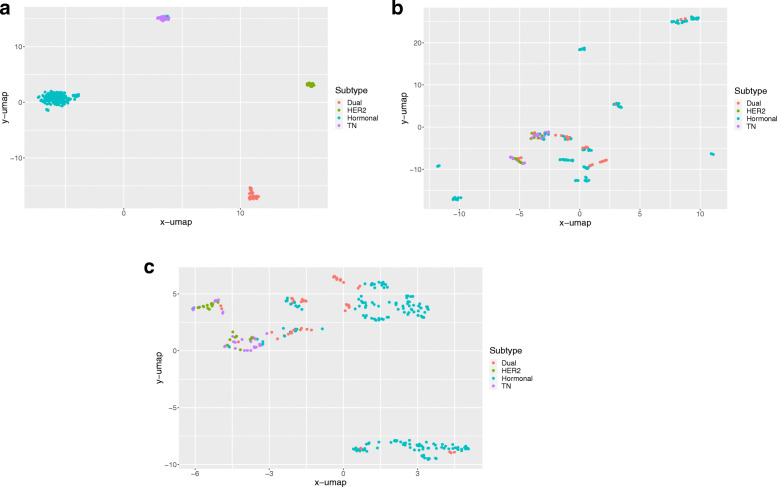
Results of supervised clustering analysis of breast cancer subtypes using different parameters. **A** ER and HER2 status, **B** ER, HER2, Ki-67, p53, EGFR, and nuclear grade, **C** ER, HER2, Ki-67, p53, EGFR, nuclear grade, and SUV

**Fig. 4:**
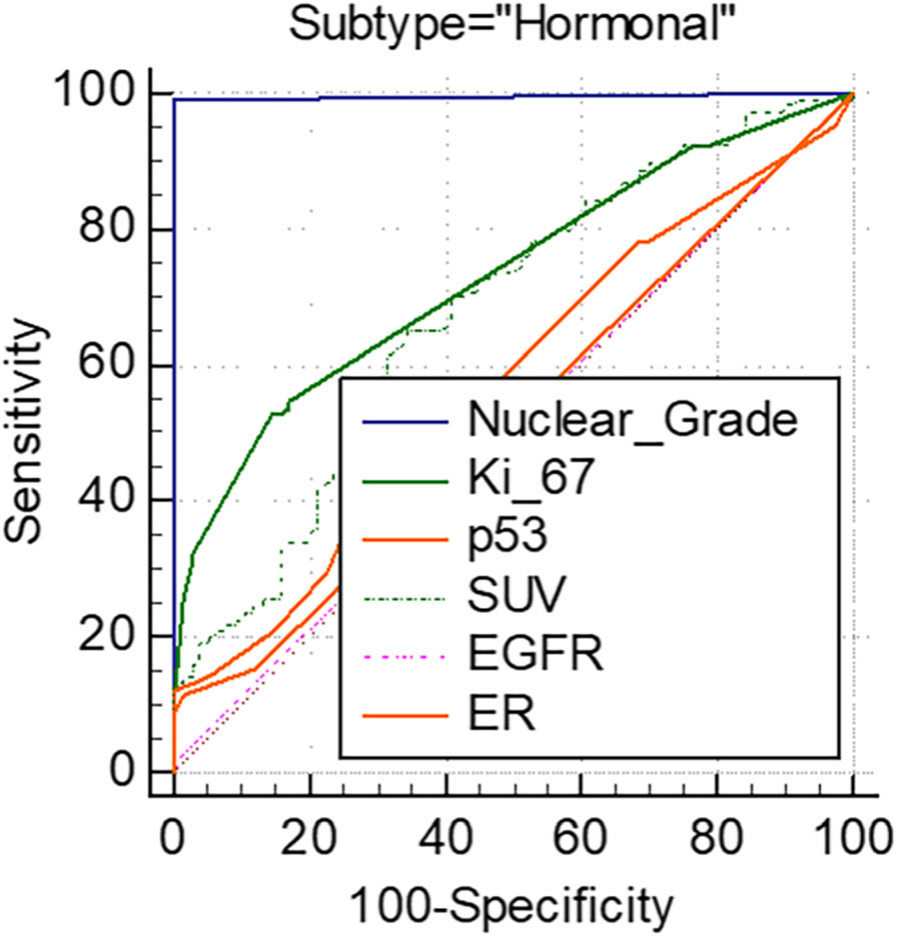
ROC curve analysis for dichotomizing the Hormonal subtype using immunohistochemical and metabolic parameters

To evaluate and compare the biology of the tumor subtypes, clustering analysis was performed using the molecular profiles (ER, HER2, Ki-67, p53, nuclear grade, and EGFR) and tumor metabolism (i.e., the SUV). As expected, classification based on ER and HER2 status perfectly grouped the subtypes (Fig. 3A). After adding the Ki-67, p53, nuclear grade, and EGFR results, heterogeneous subtype clusters were observed (Fig. 3B). The addition of metabolism information enabled different subtypes with varying aggressiveness to be estimated more accurately (Fig. 3C). Of these parameters, the nuclear grade was the most critical factor for classifying the cancer subtype (Fig. 4; Table 5).

**Table 5:** ROC curve analysis for binary classification of hormonal cancer subtype

Variable	Criteria	Sensitivity (%)	Specificity (%)	*P*	AUC
Nuclear Grade	2	99.1	100	< 0.001	0.995
Ki-67	9 %	52.8	85.5	< 0.001	0.733
p53	60 %	12.3	100	0.082	0.572
SUV	2.310	65.1	65.8	< 0.001	0.677
EGFR	positive	2.8	98.7	0.468	0.508
ER	6	11.3	98.7	0.372	0.523

## Discussion

With current advances in molecular biology that enable new therapeutic targets for breast cancer to be found, effective biomarkers have been employed to monitor therapeutic efficacy. For example, PIK3A mutations have been frequently observed in luminal A-type tumors and have emerged as a therapeutic target using PI3K inhibitors [18, 19]. Relationships between PI3K inhibitors and the disruption of glucose homeostasis suggest that PI3K inhibitors could enhance tumoral FDG uptake [20, 21]. FDG PET can provide sensitive and non-invasive information on the pharmacodynamics of PI3K inhibitors based on the PTEN status [22, 23]. The present study also revealed that the SUV was a significant factor in dichotomizing the Hormonal subtype. FDG PET could be used to evaluate the overall status of tumor glucose metabolism, which may be related to the prognosis and treatment efficacy of targeted therapies.

TN tumors showed significantly higher Ki-67 expression, p53 mutations, and nuclear grade, which are known to be associated with a worse prognosis. The hallmark of TN tumors is the overexpression of high proliferation pathways. Ki-67 has become the most widely used marker for evaluating the proliferation of breast cancer [24]. Previous studies have reported a strong correlation between tumoral FDG uptake and Ki-67 expression levels [13–15, 25]. In the present study, Ki-67 expression was significantly correlated with the SUV (Pearson’s *r* of 0.331, *p* < 0.001). It was also an independent factor for the tumor SUV. Interestingly, the mean values for Ki-67, p53, and the SUV were not significantly different according to CK5/6 positivity (*p* = 0.503, *p* = 0.913, and *p* = 0.858, respectively). Previous studies have reported that CK5/6 is positively correlated with high-grade tumors and poor survival [26, 27]. The nonsignificant difference in CK5/6 positivity between the tumor subtypes (43.4 %, 27.7 %, 62.5 %, and 55.6 % for Hormonal, Dual, HER2, and TN tumors, respectively) in this study may explain this finding.

Of the 44 EGFR-positive tumors, 23 and 12 were TN and HER2 tumors, respectively. The SUV was significantly higher for EGFR-positive tumors than for EGFR-negative tumors (4.9 vs. 3.2, *p* = 0.027). EGFR expression is one of the components of the core basal phenotype in TN tumors. It is significantly related to BRCA1 mutations [28]. Interestingly, the interaction between p53 mutations and EGFR was noted in the regression analysis for determining the SUV. p53 status and EGFR signaling can interfere with the control of the cell cycle and apoptosis. p53 mutations are known to amplify EGFR signaling in breast cancer [29, 30]. However, the effect of p53 status on EGFR-inhibitor treatment remains unclear for other cancer types [31, 32]. In addition, the effect of p53 on glucose metabolism is believed to be highly context-dependent [33]. Further research is needed to determine the mechanisms underlying the interactions between key mutations in breast cancer.

Several studies have reported the prognostic role of FDG PET in breast cancer. The change in glucose metabolism after neoadjuvant chemotherapy is a biomarker for treatment efficacy and histopathologic response. Early identification of potential non-responders to chemotherapy may be helpful in patients’ treatment decisions [34, 35]. The metabolic activity of primary breast cancer can be used as a surrogate marker for patient survival [36, 37]. Metabolic volume measured using FDG PET (which is correlated with the number of circulating tumor cells) has also been suggested to be a useful prognostic marker [38]. Non-invasive monitoring of tumoral glucose metabolism would be helpful for phenotyping tumor aggressiveness and suggesting different treatment strategies even for the same molecular subtype of breast cancer [39, 40].

Several factors should be considered when interpreting the SUV as a representative of tumor metabolism. The injected FDG dose, patient weight, time acquired, wait time before scanning, and acquisition time are closely related to each other in determining tumoral FDG uptake. For example, higher injection doses (6 to 10 MBq/kg) and/or longer acquisition times per bed are needed for patients with a large body mass [41, 42]. Guidelines have suggested a linear and a quadratic relationship between the body mass and the administered dose [43, 44]. Serum glucose levels should be checked before administering FDG and can be used to correct the SUV [45]. The recommended acquisition time is 60 min after FDG injection with an acceptable range of 55 − 75 min [46]. Efforts to maintain accurate PET image quality are also necessary in clinical practice for evaluating tumor metabolism as a characteristic of tumor biology.

Voxel-based FDG PET parameters have been used to investigate tumoral glucose metabolism. The maximum SUV, which is the highest voxel value within an ROI, is the most widely used PET parameter because of its convenient and reproducible measurement process. However, this has been known to be affected by tumor size and it is vulnerable to noise [47]. In this study, tumor size was positively correlated with the tumor SUV, which might be a confounding factor when evaluating tumor metabolism. To improve the robustness of the maximum SUV, the peak SUV has been introduced as an alternative voxel-based parameter, which is the mean SUV within a 1-cm^3^ volume of interest (VOI) including the maximum SUV. Volumetric PET parameters including the metabolic tumor volume (MTV) and total lesion glycolysis (TLG) have also taken on greater importance in prognostic prediction over SUV parameters [48, 49]. In breast cancer, the MTV may be associated with axillary lymph node metastasis and overall survival [50–52]. In addition, TLG can be used to predict event-free survival [53]. Further research evaluating the correlation between molecular characteristics and volumetric parameters is necessary for patients with breast cancer.

PET imaging techniques with novel tracers enable the visualization of IHC markers for breast cancer *in vivo*. ^18^ F-fluoroestradiol (FES) is an analog of estradiol and binds to ER in tumors and normal tissue [54, 55]. The FES uptake in breast cancer is directly correlated with ER expression, and FES PET can predict the response to hormonal therapy [56, 57]. HER2-targeting PET has been investigated for the detection of HER2-positive tumors and to predict the response to trastuzumab therapy [58]. The integration of molecular PET imaging would help to determine the subtypes of breast cancer simultaneously and predict the prognosis for patients undergoing target-driven treatment.

Therapy and intervention for tumor tissue often result in an inflammatory response, which leads to higher tumor FDG uptake. With breast cancer, fat necrosis of tissue after trauma, biopsy, surgery, or radiotherapy has been known to lead to false-positive FDG uptake [59, 60]. Previous studies using FDG positron emission mammography (PEM) have reported that a small portion (< 10 %) of cases return false-positive findings from areas affected by fat necrosis or an excisional biopsy [61, 62]. In this study, FDG PET/CT scan was conducted 10.5 ± 5.9 days after the breast biopsy. Although factors affecting FDG uptake in tumors such as a previous biopsy are recognized to accurately interpret PET images in clinical practice, these are problematic in quantitative analysis. A sub-optimized protocol for the control of the time interval between a biopsy and FDG PET imaging in the current retrospective study may be a limitation for the measurement of tumoral glucose metabolism [63].

In this study, clustering analysis was conducted based on immunohistochemical markers and tumor metabolic characteristics. Though precision medicine based on comprehensive molecular biomarkers is accessible for breast cancer treatment, the improvement of diagnostic systems using cost-effective basic clinicopathologic parameters is required for patient management [64]. Tumoral metabolic information provided by FDG PET/CT enables cancer subtypes to be clustered successfully and raises the possibility of classifying aggressive tumors from among traditionally determined subtypes. Of the various methods available for clustering analysis, UMAP, which was used in this study, has the advantages of effective dimensional reduction while preserving meaningful distance information between clusters [17]. However, the prognostic implications of combining molecular and metabolic information need to be investigated further.

Although we have shown that useful information for patient management can be obtained by combining metabolic parameters from FDG PET and pathological classification, it is important to note that our study has several limitations. For example, we describe a retrospective study at a single institution, which may have led to possible selection bias. In addition, our cancer typing was based on immunohistochemistry rather than gene expression-based assays, but pragmatically this is the method utilized in most centers when classifying breast cancer. Our follow-up period also did not allow for the robust collation of outcome data, such as disease-free status and overall survival, which will be of interest in future studies.

## Conclusions

The metabolic activity of breast cancer measured using FDG PET/CT was significantly correlated with molecular alteration profiles as assessed using immunohistochemical data. Combining molecular markers and metabolic information may assist in the understanding of tumor aggressiveness in breast cancer. Further investigations are needed to determine the relationship between the molecular landscape and clinical metabolic phenotypes so that appropriate therapeutic strategies can be developed to improve patients’ prognoses.

## Supplementary information

**Additional file 1.**

**Additional file 2.**

## Data Availability

The datasets used and/or analyzed during the current study are available from the corresponding author on request.

## Abbreviations

CK: cytokeratin
DCIS: ductal carcinoma in situ
EGFR: epidermal growth factor receptor
ER: estrogen receptors
FDG: ^18^ F-fluorodeoxyglucose
FES: ^18^ F-fluoroestradiol
HER2: human epidermal growth factor receptor 2
IHC: immunohistochemistry
MTV: metabolic tumor volume
PEM: positron emission mammography
PET/CT: positron emission tomography/computed tomography
PR: progesterone receptors
ROI: region of interest
SUV: maximal standardized uptake value
TLG: total lesion glycolysis
TN: triple negative
UMAP: Uniform Manifold Approximation and Projection
VOI: volume of interest
