# Supplementary figures and images for: Clustering subtypes of breast cancer by combining immunohistochemistry profiles and metabolism characteristics measured using FDG PET/CT
Source: Cancer Imaging. 2021 Sep 27;21:55. doi: 10.1186/s40644-021-00424-4 (PMC8477513; doi:10.1186/s40644-021-00424-4)

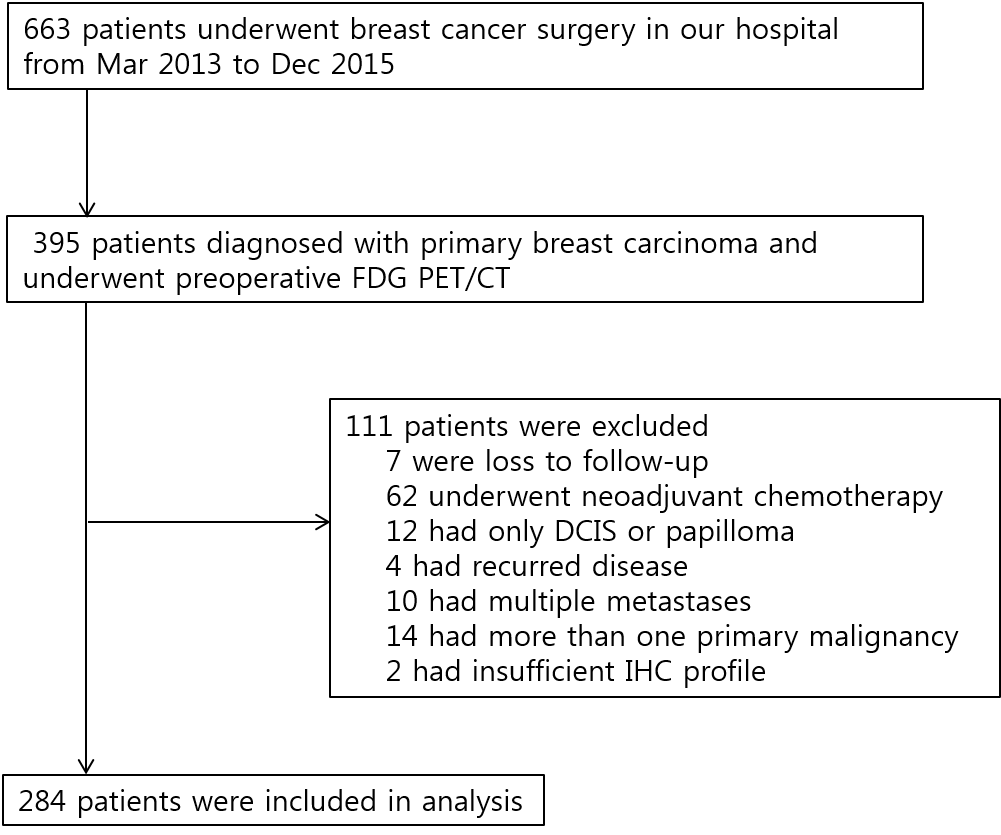

Supplement: Supplementary file 1 — Additional file 1. [file 40644_2021_424_MOESM1_ESM.tif]
