# Supplementary material for: Clustering subtypes of breast cancer by combining immunohistochemistry profiles and metabolism characteristics measured using FDG PET/CT
Source: Cancer Imaging. 2021 Sep 27;21:55. doi: 10.1186/s40644-021-00424-4 (PMC8477513; doi:10.1186/s40644-021-00424-4)
Supplement: Supplementary file 2 — Additional file 2. [file 40644_2021_424_MOESM2_ESM.docx]

Supplementary Table 1. Histopathologic tumor characteristics of 111 excluded cases

| Characteristics | (% or mean ± SD) |
| --- | --- |
| Gross histopathology |  |
| Invasive ductal carcinoma^*^ | 90.1% |
| Invasive lobular carcinoma | 5.4% |
| Papillary carcinoma | 1.8% |
| Medullary carcinoma | 0.9% |
| Neuroendocrine carcinoma | 0.9% |
| Positive ER | 43.2% |
| Positive PR | 43.2% |
| Positive C-erbB2 (≥ 2) | 29.7% |
| Ki-67^†^ | 36 ± 25.6 |

ER, estrogen receptor; PR, progesterone receptor; ^*^including high-grade ductal carcinoma in situ, ^†^upper bound value of positive cell population (%)
